# Neuroticism Modulates the Functional Connectivity From Amygdala to Frontal Networks in Females When Avoiding Emotional Negative Pictures

**DOI:** 10.3389/fnbeh.2019.00102

**Published:** 2019-05-09

**Authors:** Yaling Deng, Shijia Li, Renlai Zhou, Martin Walter

**Affiliations:** ^1^Neuroscience and Intelligent Media Institute, Communication University of China, Beijing, China; ^2^Key Laboratory of Brain Functional Genomics (MOE&STCSM), Shanghai Changning-ECNU Mental Health Center, School of Psychology and Cognitive Science, East China Normal University, Shanghai, China; ^3^Shanghai Key Laboratory of Magnetic Resonance, East China Normal University, Shanghai, China; ^4^Department of Psychology, School of Social and Behavior Sciences, Nanjing University, Nanjing, China; ^5^Department of Psychiatry and Psychotherapy, Eberhard Karls University, Tübingen, Germany; ^6^Max Planck Institute for Biological Cybernetics, Tübingen, Germany; ^7^Department for Behavioral Neurology, Leibniz Institute for Neurobiology, Magdeburg, Germany; ^8^Clinical Affective Neuroimaging Laboratory (CANLAB), Otto-von-Guericke University, Magdeburg, Germany

**Keywords:** functional connectivity, neuroticism, motivational direction, dorsomedial prefrontal cortex, middle cingulate cortex

## Abstract

Amygdala activity was previously found to correlate with neuroticism as an effect of valence, but so far few studies have focused on motivational context. The network subserving altered amygdala activity has not yet been investigated although some studies showed strong effective connections with prefrontal cortex (PFC). The goal of this study was to test the modulatory role of neuroticism on the functional connectivity (FC) between amygdala and other brain regions, especially PFC, during emotion processing from motivational direction. We applied an emotional picture viewing paradigm with different motivational directions (approaching and avoiding) in a large participant sample. The results showed that neuroticism predicted the amount of amygdala FC to dorsomedial PFC (dmPFC) and middle cingulate cortex (MCC). Increased FC during negative vs. positive pictures was found primarily in low neuroticism subjects, especially during the avoid condition. This valence and motivation dependent connectivity increase were disrupted for high neurotic participants. No effect of neuroticism was found for the approach condition. We showed that neuroticism, especially in the context of passive affect regulation, may have impaired connectivity between amygdala and putative regulatory cortical networks.

## Introduction

Neuroticism is one of the Big Five dimensions of personality traits (Digman, 1990) and individuals who score high on neuroticism showed a strong tendency to experience negative affection, such as anxiety, worry, fear, anger, frustration, envy, jealousy, guilt, depressive mood and loneliness, especially when threatened, frustrated, or facing loss (Thompson, 2008; for review, see Ormel et al., 2013a). It is important to identify and map the underlying neurobiological correlates of neuroticism, because people with high neuroticism are often at risk of many forms of psychopathology and behavioral problems, especially the common mental disorders, such as mood disorders and substance abuse (for review, see Lahey, 2009; Kotov et al., 2010; Ormel et al., 2013b; Jeronimus et al., 2016).

Functional magnetic resonance imaging (fMRI) studies revealed the critical function of limbic system in influencing the neuroticism, such as the amygdala, the hippocampus, as well as the frontal regions, e.g., the anterior cingulate cortex (ACC) and medial prefrontal cortex (mPFC; Canli, 2008; Tzschoppe et al., 2013; Aghajani et al., 2014; Everaerd et al., 2015; also reviewed in Servaas et al., 2013). Among those brain regions, amygdala is the key region that is involved in emotional procession and emotional responses (Kim et al., 2011), especially the cortex-amygdala integrated system (see a very recent review Pessoa, 2017). Studies have found that neuroticism is positively related to increased amygdala activity (Haas et al., 2007; Canli, 2008; Chan et al., 2009; Cremers et al., 2010), while some others found no such correlation (Mobbs et al., 2005; Drabant et al., 2009; Cremers et al., 2010; Hyde et al., 2014). It is possible that the inconsistence of amygdala involvement in regulating neuroticism is highly dependent on the task type: amygdala might be highly involved in emotion and stress-related tasks (e.g., Tzschoppe et al., 2013; Everaerd et al., 2015), but less involved in decision making and neutral tasks (e.g., Forbes et al., 2014; Szameitat et al., 2016).

Nevertheless, further investigation on the neuroticism related neural activity is still needed, especially the functional connectivity (FC) between amygdala and other brain networks. For instance, the amygdala-frontal circuity, is crucial for the integration between emotion and cognition (Cremers et al., 2010; Tzschoppe et al., 2013; also reviewed in Pessoa, 2017), and the disconnection between amygdala and frontal regions (such as ACC, mPFC) may play an important role in the elevated emotional reaction to negative stimuli in the high neurotic individuals (Cremers et al., 2010; Xu and Potenza, 2012; Bjørnebekk et al., 2013; Ormel et al., 2013b). For instance, Wang et al. (2018) using resting state fMRI found that neuroticism was positively correlated with altered functional coupling of the basolateral amygdala with the anterior insular cortex, dorsal and pregenual ACC. Cremers et al. (2010) reported that higher neurotic individuals showed stronger right amygdala to dorsomedial PFC (dmPFC) FC for negative (angry and fearful) compared to neutral faces; while higher neurotic individuals showed weaker left amygdala to ACC FC for negative (angry, fearful and sad) compared to neutral faces. An aversive learning study using unpleasant sound found that higher neurotic individuals were associated with a stronger interaction between the right amygdala and the right hippocampus, as well as the right amygdala and prefrontal cortical regions, especially ventromedial PFC (vmPFC), dorsolateral PFC (dlPFC), and ACC (Tzschoppe et al., 2013). Kruschwitz et al. (2015) investigated 178 subjects and revealed that higher neurotic individuals showed stronger bilateral amygdala resting-state FC to the fusiform regions, a region that was crucial for the face processing (Halgren et al., 1999); and evidence showed that neuroticism may impact fusiform gyrus activity in response to emotional facial expressions (Chan et al., 2009).

Taken together, disconnection between the amygdala and other brain regions may underlie the observed heightened emotional reactivity to negative events in higher neurotic individuals (Perlman et al., 2009). On one hand, the FC between amygdala and hippocampus or amygdala and fusiform region in higher neurotic individuals may be more involved in the aversive leaning system and emotional facial processing system than lower neurotic individuals. On the other hand, higher neurotic individuals may be linked with weaker amygdala and frontal cortex FC therefore showed abnormal cognitive control abilities. Taken together, these FCs may result in the negative bias in higher neurotic individuals, therefore related to high risk of mood disorders (Servaas et al., 2013).

Previous studies that focused on the connection between amygdala FCs and emotional processing used mostly the valence or arousal dimensions of emotion; however, it is still unclear whether amygdale FCs were regulated by emotional motivation as well, such as approach or avoid (Lang et al., 1998). Changes in emotional motivation direction may change the affective meaning of a stimulus; for example, approaching the negative stimulus increased the unpleasant feelings of participants, but avoiding negative stimulus released such unpleasant feelings (Cunningham et al., 2008). Furthermore, Cunningham et al. (2008) proposed the motivational salience hypothesis of amygdala, which states that the amygdala is sensitive to motivational relevance in addition to valence. According to their hypothesis, amygdala signals the important information that related to challenges, threats or opportunities in the current situation, therefore modulating the appropriate processes such as perceptual, attention, and cognitive. To gain a better understanding of the neural basis of emotion processing dimensions related to neuroticism, motivational direction of emotion is therefore required. To our knowledge, so far no studies have systematically investigated the network subserving altered amygdala activity from motivational direction as well as emotional valence, which provided us with a good reason to run the current experiment.

Our goal was to test the modulatory role of neuroticism on the FC between amygdala and other brain regions, especially PFC, during emotion processing from both valence and motivational directions. We applied an emotional picture viewing paradigm with different motivational directions (approaching and avoiding) in a large participant sample. Based on the previous studies that higher neurotic individuals showed biased negative emotion processing (Haas et al., 2007; Servaas et al., 2013), we hypothesize that high neurotic individuals may have impaired cognitive control so their FC between the amygdala and PFC might show a stronger disconnection for negative stimulus. Besides, changes in motivational direction may influence the FCs and the higher neurotic individuals may show a stronger reaction when avoiding negative stimulus, so the individual differences in neuroticism of amygdala to PFC FC should vary in motivational direction, especially in the avoiding condition.

## Materials and Methods

### Participants and Ethics

We recruited volunteers *via* advertisement on the Internet. One-hundred and eighty-five volunteers applied for participating in this study. We first assessed whether each participant met the criteria of this study. Exclusion criteria (as assessed by self-report and physical examination) includes a personal history of diagnosed psychopathology, substance abuse during 6 months prior to the scan, neurological problems, current use of mood-altering medication and oral contraceptive intake, pregnancy or any standard MRI counter indications. All participants provided written informed consent. To avoid the influence of menstrual cycle, all the participants carried out the experiment during their luteal phase according to their self-report.

To access neuroticism traits, all participants completed the Eysenck Personality Questionnaire-Short Scale for Chinese (Qian et al., 2000). Participants whose neuroticism scores were lower than Mean − one SD were sorted into the low neuroticism group, while scores higher than mean + one SD were sorted into the high neuroticism group. Finally, 50 healthy right-handed female participants took part in the present study. The mean age of participants was 23.47 years old (SD = 3.42, range: 18–32 years old). Twenty-six participants were grouped as high neuroticism (hN, Mean = 8.69, SD = 1.46) and 24 participants as low neuroticism (lN, M = 0.75, SD = 1.54). The neuroticism score was significantly different between the two groups (*t*_48_ = 18.702, *p* < 0.001).

The experimental procedures were approved by the Institutional Review Board of the State Key Laboratory of Cognitive Neurosciences and Learning of Beijing Normal University. All the participants gave written informed consent before participating.

### Materials and Experimental Design

During fMRI scanning, participants were presented with pictures selected from International affective picture system (IAPS; Lang et al., 2005). In total, 72 positive, 72 negative and 72 neutral pictures were selected and matched on normed official Chinese ratings of arousal (*M*_positive_ = 6.00, *SD*_positive_ = 0.73; *M*_negative_ = 5.70, *SD*_negative_ = 0.70; *M*_neutral_ = 4.37, *SD*_neutral_ = 0.52) and valence extremity (*M*_positive_ = 7.40, *SD*_positive_ = 0.45; *M*_negative_ = 2.06, *SD*_negative_ = 0.49; *M*_neutral_ = 4.84, *SD*_neutral_ = 0.40). The three types of pictures were significantly different on valence (*F*_(2, 215)_ = 2581.65, *p* < 0.001) and arousal (*F*_(2, 215)_ = 126.23, *p* < 0.001). The arousal of positive and negative pictures had no difference but was significantly higher than neutral ones (both *p* < 0.001).

We used a well-established paradigm in the present experiment (Cunningham et al., 2011). Pictures were presented one each at a time, and participants were instructed to press one button to initiate either an “approach” or “avoid” condition. More specifically, all pictures were initially presented to fill 75% of the screen (in the middle). During the “approach” condition, when participants pressed the button, the picture would expand until it filled 100% of the screen, in order to simulate the condition that the participants were moving towards the picture. During the “avoid” condition, when participants pressed the button, the picture would contract until it filled 50% of the screen, in order to simulate the condition that the participants were moving away from the picture. Participants were instructed to imagine that they were approaching or avoiding the scene in the pictures as it grew or shrank. The pictures changed at 5% to create fluid motion and took 3 s to reach 100% or 50% of the screen, respectively.

Participants were instructed to press only one button in each block of trials as soon as the picture appeared, to create a motivational frame and to ensure that participants equally approached and avoided all stimuli. There were three runs, and 12 blocks per run, for a total of 36 blocks (18 blocks during each condition). Each block contained six trials and in total there were 216 trials. Positive, negative and neutral pictures were equally distributed and randomly displayed in each block, whereas approach and avoid blocks were also equally distributed and randomly distributed in each run. Instructions lasted 4 s prior to each block to indicate whether participants were to approach or to avoid forthcoming stimuli in this block. A fixation cross appeared for 4 s after the instructions, and a variable fixation of 2 s, 4 s, or 6 s appeared between each picture to allow the estimation of the hemodynamic response.

### Image Acquisition and Analysis

Images were acquired on a Siemens Prisma 3T MR-scanner, flip angle 90° using axial whole-brain acquisition, with an interleaved slice acquisition order (33 slices, slice thickness = 3.5 mm, TR = 2,000 ms, TE = 30 ms, voxel size = 3.5 × 3.5 × 4.2 mm, matrix size = 64 × 64, FOV = 224 × 224). Before functional imaging, a high resolution T1 anatomical image (slice thickness = 1 mm, TR = 2,530 ms, TE = 2.98, FOV = 224 × 256) was collected for normalization.

#### Preprocessing

Functional data were preprocessed and analyzed using the statistical parametric mapping software package SPM 12^1^ implemented in Matlab 12a^2^.

The EPI volumes were reoriented with respect to the anterior commissure selected on the first volume. Time series were corrected for differences in slice acquisition times. After spatial realignment to the first image, the movement parameters for each participant were inspected: if a participant moved more than 3 mm in any direction (anterior-posterior, right-left, inferior-superior), the data were excluded from further analysis. For each participant, functional EPI scans were co-registered to their corresponding high-resolution T1 anatomical image. The T1 images were spatially normalized to the SPM12 MNI template. The transformations from the co-registration and normalization steps were applied to the EPI functional scans and new images were created and re-sized to 3 × 3 × 3 mm. To enhance signal-to-noise ratios, these images were smoothed using an 8 mm full-width-half-maximum (FWHM) kernel.

Low-frequency noise was removed by applying a high-pass filter (cut-off of 128 s) to the fMRI time series at each voxel and modeled for temporal autocorrelation across scans with an autoregressive AR(1) model. Significant hemodynamic changes for each condition were calculated using the general linear model (Friston et al., 1997), with respect to the event-related response convolved with canonical hemodynamic response function. First level effects were modeled by convolving an event-related hemodynamic response function and its temporal derivative against the preprocessed data for each of the six conditions (1 - approach positive, 2 - approach negative, 3 - approach neutral, 4 - avoid positive, 5 - avoid negative, 6 - avoid neutral).

In order to run the follow-up FC analysis, we modeled three motivation-independent contrasts: (1) Negative vs. Positive; (2) Negative vs. Neutral; and (3) Positive vs. Neutral, and six motivation-dependent contrasts: (1) Approach: Negative vs. Positive; (2) Approach: Negative vs. Neutral; (3) Approach: Positive vs. Neutral; (4) Avoid: Negative vs. Positive; (5) Avoid: Negative vs. Neutral; and (6) Avoid: Positive vs. Neutral. Two-sample *t*-tests were calculated for the group effects (high vs. low neurotic) and the significance threshold was set as cluster-level *p* < 0.05 family-wise error (FWE) correction. In order to check the task-related amygdala activation, we performed the region of interest (ROI) analysis and restricted the search areas within bilateral amygdala. The left and right amygdala masks were created by the WFU pickatlas toolbox (Maldjian et al., 2003) and then used for the ROI analysis. The initial threshold was set as *p* < 0.05 uncorrected, and the results survived peak-level *p* < 0.05 FWE correction were reported as significant results.

#### Psycho-Physiological Interaction Analysis

Previous studies indicated that the amygdala is correlated with emotion and neuroticism (Kim et al., 2011), therefore, in order to investigate whether the amygdala-based FCs were regulated by emotional valence and motivation processing, and we applied psycho-physiological interaction (PPI) analysis (Friston et al., 1997). The PPI analysis employed a design matrix with three regressors: (i) the “psychological variable” representing the cognitive process of interest; (ii) the “physiological variable” representing the neural response in the seed region; and (iii) the interaction term of (i) and (ii). We first limited the regions of interest within bilateral amygdala masks that predefined in ROI analysis and then extracted the individual time series from a 6 mm radius sphere that centered at the peak coordinates within the amygdala masks. Two sample *t*-tests were used to reveal the group FC differences between high and low neurotic individuals in these contrasts: (1) Negative vs. Positive; (2) Negative vs. Neutral; (3) Positive vs. Neutral; (4) Approach: Negative vs. Positive; (5) Approach: Negative vs. Neutral; (6) Approach: Positive vs. Neutral; (7) Avoid: Negative vs. Positive; (8) Avoid: Negative vs. Neutral; and (9) Avoid: Positive vs. Neutral. The significance threshold used for PPI analyses was cluster-level *p* < 0.05 FWE correction.

## Results

### Behavioral Results

**Figure 1:**
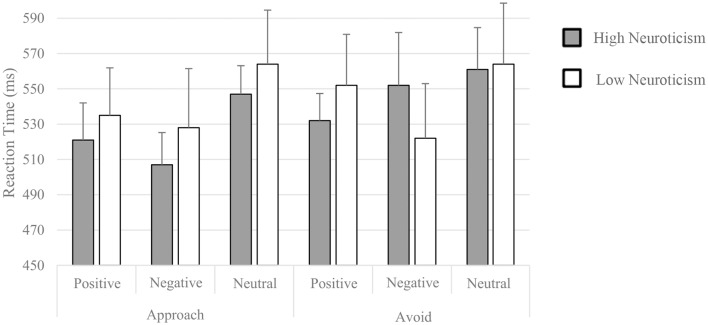
Mean reaction time (RT) for each condition of two groups.

The mean reaction time (RT) for all conditions in each group was shown in Figure 1. Three outliers were excluded. We applied a 2 (within-subject factor motivation: approach, avoid) × 3 (within-subject factor valence: positive, negative, neutral) × 2 (between-subject factor neuroticism: high, low) mixed measurement variance analysis to check the main effects and interactions. We found a main effect of valence (*F*_(2,90)_ = 5.409, *p* = 0.006), driven by a faster RT for negative (*t*_46_ = 3.460, *p* = 0.004) and positive pictures (*t*_46_ = 2.968, *p* = 0.014) compared to neutral pictures. We also found a main effect of motivation (*F*_(1,45)_ = 3.698, *p* = 0.061), driven by a faster RT for approach condition. Main effect of neuroticism (group) was not significant. No significant interactions were found here.

### PPI Results

#### Motivation-Independent FCs

**Figure 2:**
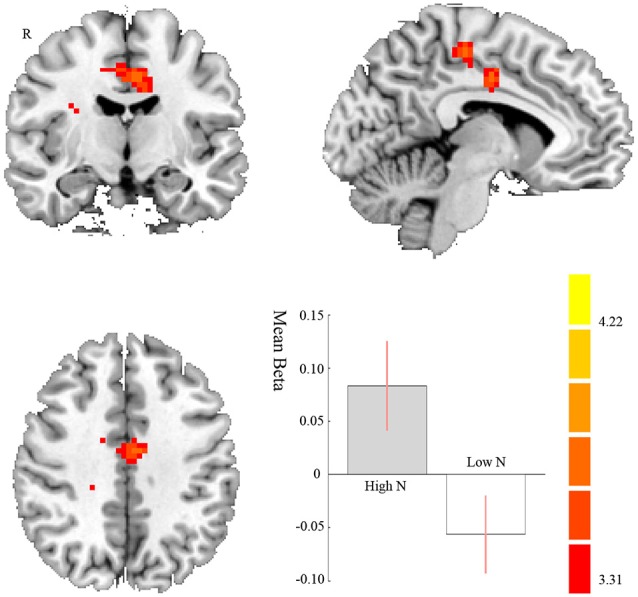
Brain regions displaying differences between high and low neurotic individuals of the functional connectivity (FC) with right amygdala and right middle cingulate cortex (MCC) for negative compared to positive pictures. The contrast estimate of 90% confidence intervals is shown. The color bar represents the *t*-values.

We found a significant stronger FC between right amygdala and right middle cingulate cortex (MCC; *t*_42_ = 4.13, *p* = 0.027 FWE correction; MNI: 18, −16, 41; cluster size = 153) when processing the positive pictures compared to negative pictures, which is more evident in high neurotic individual than low neurotic individuals. In other words, high neurotic individuals had significantly stronger disconnection between right amygdala and right MCC when processing the negative pictures (see Figure 2, Table 1). We did not find any significant results on Negative vs. Neutral and Positive vs. Neutral contrasts.

**Table 1:** The differences between high and low neuroticism individuals of the functional connectivity (FC) with the right and left amygdala for different contrasts.

Seed region	Contrast	Brain regions	Side	Cluster size	*T*-values	*P*-values	MNI coordinates		
							*x*	*y*	*z*
Right amygdala	Negative < Positive	MCC	R	153	4.13	0.027	18	−16	41
Right amygdala	Avoid: Negative < Positive	dmPFC	R&L	389	5.20	0.000	−6	50	35
		MCC and premotor SMA	R	232	4.10	0.006	15	−28	62
Left amygdala	Avoid: Negative < Neutral	MCC	R&L	153	3.79	0.049	−3	−10	38

#### FC Changes Only Evident in Avoid Situation

The whole-brain and ROI analysis did not reveal any significant difference between two groups in the activation of amygdala. Then we performed PPI analysis and the coupling between amygdala and several other regions were sensitive to motivation and valence interaction.

**Figure 3:**
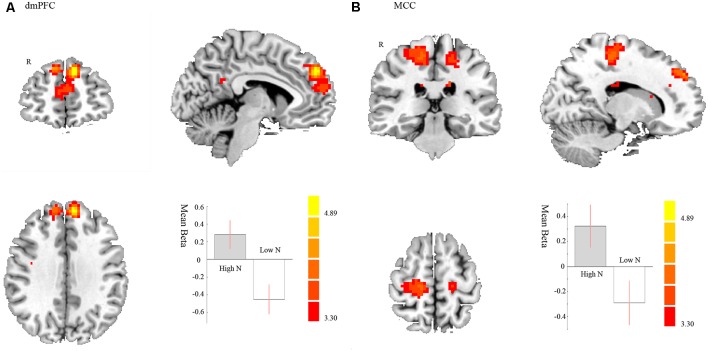
Negative < Positive in avoiding condition. Brain regions displaying differences between high and low neurotic individuals of the FC with right amygdala and **(A)** dorsal medial prefrontal cortex (dmPFC) and **(B)** right MCC and premotor supplementary motor area (SMA) for negative compared to positive pictures in avoiding condition. The contrast estimate of 90% confidence intervals is shown. The color bar represents the *t*-values.

In the avoiding condition, for the negative < positive contrast, we found a stronger FC between the right amygdala and dmPFC in high compared to low neurotic individuals (*t*_42_ = 5.20, *p* < 0.001 FWE correction; MNI: −6, 50, 35; cluster size = 389). That is to say, high neurotic individuals had a stronger disconnection between right amygdala and dmPFC when avoiding the negative pictures (see Figure 3A, Table 1). We also found a stronger FC between the right amygdala and the right posterior dorsomedial frontal cortex [including the right MCC and premotor supplementary motor area (SMA)] on high compared to low neurotic individuals (*t*_42_ = 4.10, *p* = 0.006 FWE correction; MNI: 15, −28, 62; cluster size = 232, see Figure 3B, Table 1).

**Figure 4:**
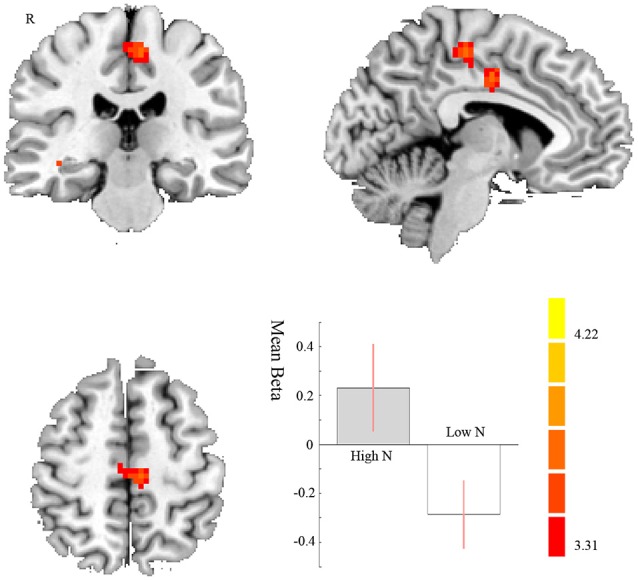
Negative < Neutral in avoiding condition. MCC displaying differences between high and low neurotic individuals of the FC with left amygdala for negative compared to neutral pictures in avoiding condition. The contrast estimate of 90% confidence intervals is shown. The color bar represents the *t*-values.

In avoiding condition, for the negative < neutral contrast, we found a stronger FC between the left amygdala and MCC on high compared to low neurotic individuals (*t*_39_ = 3.79, *p* = 0.049 FWE correction; MNI: −3, −10, 38; cluster size = 153). This indicates that high neurotic individuals had stronger disconnection between left amygdala and right MCC when avoiding the negative pictures (see Figure 4, Table 1).

We did not find any significant results on Positive vs. Neutral contrast. Besides, it is important to note that there were no significant differences between high and low neurotic individuals when approaching pictures, so the main effect of the disconnection between right amygdala and MCC and dmPFC when processing negative pictures for high neurotic individuals is avoiding pictures.

## Discussion

The main purpose of this study was to test the modulatory role of neuroticism on the FC between amygdala and other brain regions, especially PFC, during emotional approach/avoid processing. This is the first FC study about neuroticism that takes the motivational direction of emotion into consideration and the results showed the disconnection between amygdala and dmPFC, MCC and premotor SMA in high neurotic individuals when processing negative stimulus. More importantly, this effect was mainly driven by avoiding condition.

### Neuroticism Modulates Amygdala-MCC Connectivity

We found that high neurotic individuals showed stronger disconnection between amygdala and MCC when processing the negative pictures, rather than positive or neutral pictures. And this effect was most evident in the avoiding condition.

MCC has been associated with negative emotion processing, such as fear (Vogt et al., 2003) and sadness (reviewed in Phan et al., 2002); and is activated during a cognitive appraisal condition of negative stimulus (Taylor et al., 2000). Frank et al. (2014) even found increased MCC activation in emotional regulation, especially during upregulation. The motivation independent results suggest that the higher neurotic individuals showed stronger negative emotional processing and appraisal.

Besides, the posterior medial frontal cortex, including the MCC and SMA, is known to act as the “interface” between the limbic system (emotion) and motor system (action; Oliveri et al., 2003); and is well-recognized as a “performance monitoring” region. Potentially it has the function of action preparation driven by emotion (Dum and Strick, 1991; Shima et al., 1991; Morecraft and van Hoesen, 1992; Ridderinkhof et al., 2004). The FC between the SMA and other brain areas that are responsive to emotion in others reflects an empathic response to negative emotional stimuli (Pratumvinit et al., 2016). A recent study found increased FC between SMA and salience network during high salience expectancy (expecting highly arousing pictures) reflecting internal preparation to react to emotionally salient stimuli (Li et al., 2017). In the present study, we found significant amygdala to MCC/premotor SMA FC between high/low neurotic groups, providing a behavioral relevance in the context of motor preparation (Murase et al., 2005): high neurotic individuals showed stronger disconnection between amygdala and MCC-premotor SMA in avoiding negative stimuli rather than approaching negative stimuli. Based on these findings, we proposed that when passively “avoiding” aversive stimuli, higher neurotic individuals engaged less emotion to motor system interaction, which might suggest an insufficient/impaired reaction during avoiding condition. The RT in higher neurotic individuals is relatively longer when avoiding negative stimuli, which might support the hypothesis—however, note that there is no significant correlation between the FCs and the RT.

### Neuroticism Modulates Amygdala-dmPFC Connectivity

We found that higher neurotic individuals showed stronger disconnection compared to lower neurotic individuals between right amygdala and dmPFC when avoiding the negative pictures.

Dorsal medial PFC (dmPFC) is crucial for emotional regulation (Ochsner and Gross, 2005). Emotion regulation represented how top-down and bottom-up processes compete and interact to produce optimal (or counterproductive) behavioral outcomes (Kim et al., 2011). In the emotional regulation process, the PFC controls the amygdala in response to an emotional challenge (Davidson et al., 2000). Many functional neuroimaging studies have found that there were large overlaps between PFC and amygdala activity during the successful emotional regulation process (Ochsner et al., 2002); specifically, during emotional regulation the PFC activity often increased and amygdala activity decreased (Hariri et al., 2000; Delgado et al., 2008). In general, a more efficient crosstalk between the amygdala and the PFC—usually including both medial and lateral PFC—begets a better ability to regulate one’s emotions.

In this study, when processing the negative pictures, high neurotic individuals showed a stronger disconnection between dmPFC and amygdala. A recent clinical study found that the major depressive disorder patients (the higher neurotic individuals showed potential risk of depression) showed abnormal dmPFC activation when expecting positive rather than neutral pictures (Zhang et al., 2017). Our results indicated that the high neurotic individuals’ dmPFC may provide insufficient cognitive control over amygdala when processing the negative pictures. Since dmPFC is responsible for emotional regulation, the low capacity of emotional regulation in higher neurotic individuals that related to more intensive negative emotion may be a result of the amygdala to dmPFC disconnection.

Although we found that neuroticism modulated the FC between amygdala-dmPFC and amygdala-MCC, it is important to note that FC between other brain regions also plays roles in neuroticism. For example, Hsu et al. (2018) used connectome-based predictive modeling (CPM) to resting state FC and found that the best predictive models of neuroticism from FC span the whole brain. Besides, the results in this study were different from results about resting state FC. Wang et al. (2018) used resting-state fMRI and found that higher neurotic individuals showed altered functional coupling of the basolateral amygdala with the anterior insular cortex, dorsal and pregenual ACC following acute stress exposure. The different experiment design may explain the different results. Combining present study with previous studies, it may suggest a complex and abnormal brain network relates to neuroticism, and different networks were recruited to different tasks.

### Neuroticism Modulates FC Only in Avoiding Condition

Previous studies reported a relationship between neuroticism and enhanced amygdala activity in response to negative stimulus (e.g., Servaas et al., 2013). However, in the present study, we did not find the valence-dependent group differences regardless of motivation both for the whole-brain analysis nor ROI analysis. The inconsistency between this study and the previous studies may be mainly explained by the different experiment procedure. In this study, the emotional pictures were not showing immobile as the most previous studies did. Instead, participants needed to press a button to approach or avoid the emotional pictures as them expand or contract. The input of the variable “motivation” made the psychological process more complicated. More importantly, the FC differences between high and low neurotic individuals were only found in avoiding negative condition not approaching negative condition, which fits well to our hypothesis.

In psychological terms, instinctive reactions to negative stimulus and subsequent regulatory responses are often referred to as bottom-up, emotion generation system and top-down, emotion regulation system, respectively (Kim et al., 2011). Approaching negative pictures may be the case that the initial bottom-up reactions are too intensive and exaggerated for both high and low neurotic individuals and therefore no significant group difference was found. Alternatively, avoiding negative pictures is relatively less threatening compared to approaching negative stimulus. The low neurotic individuals had normal emotional regulation of dmPFC and MCC over amygdala in this condition. But the high neurotic individuals failed to employ a top-down control mechanism, which allowed initial bottom-up responses to intrude on normal cognitive functioning. Previous study also found that high neurotic individuals have increased attention to negative stimuli (Haas et al., 2007). So when asked to avoid negative stimuli, they showed disconnection between amygdala and dmPFC and MCC. They had difficulties involving the dmPFC and MCC to gain cognitive control over avoiding negative stimulus. This result may show an impaired harm avoidance system and evidence for the fact that high neurotic individuals have increased attention to negative stimuli.

This also reflected the inflexible motivational system of high neurotic individuals. When approaching emotional pictures, high neurotic individuals could mobilize relevant brain regions to accomplish the task as good as low neurotic individuals. But when avoiding the pictures, especially negative pictures, high neurotic individuals failed to do so. Many studies measured the resting state frontal alpha wave asymmetry scores and found that high neurotic individuals showed lower relative left prefrontal activity than low neurotic individuals (e.g., Minnix and Kline, 2004; Huang et al., 2015). Individuals who had relative right asymmetry during rest showed lower emotional flexibility while processing emotional stimulus than individuals with relative left asymmetry (Liu et al., 2014). Our results gave further neurological suggestion that high neurotic individuals had an inflexible emotional reactive system.

### Limitations

In the current study, we only used female participants. These results cannot be generalized to the entire population because many researchers have shown that females have higher level of neuroticism than males (Costa et al., 2001). Functional MRI studies also found an opposite pattern of brain activation in females compared to males (Merz et al., 2010). Clearly, more detailed and systematic research is deserved to investigate the sex differences in neuroticism to get a better understanding of neurobiological basis of neuroticism.

## Conclusion

Our present study indicates that neuroticism is an important modulation of amygdala-dmPFC FC and amygdala-MCC FC when processing negative stimulus, especially in the avoiding condition. We could show that neuroticism, especially in the context of passive emotional regulation, had impaired connectivity between amygdala and putative regulatory cortical networks. These findings may explain why high neuroticism individuals had more negative emotion and the neural mechanisms associated with the development of affective disorders.

## Ethics Statement

The experimental procedures were approved by the Institutional Review Board of the State Key Laboratory of Cognitive Neurosciences and Learning of Beijing Normal University. All the participants gave written informed consent before participating.

## Author Contributions

All authors listed have made a substantial, direct and intellectual contribution to the work, and approved it for publication.

## Conflict of Interest Statement

The authors declare that the research was conducted in the absence of any commercial or financial relationships that could be construed as a potential conflict of interest.

## Footnotes

^1^http://www.fil.ion.ucl.ac.uk/spm/

^2^www.mathworks.com/products/matlab/
